# Usefulness of reinforcing interventions on continuous positive airway pressure compliance

**DOI:** 10.1186/1471-2466-14-78

**Published:** 2014-05-03

**Authors:** Anna Lo Bue, Adriana Salvaggio, Serena Iacono Isidoro, Salvatore Romano, Oreste Marrone, Giuseppe Insalaco

**Affiliations:** 1 National Research Council of Italy, Institute of Biomedicine and Molecular Immunology “A. Monroy”, Via Ugo La Malfa, 153, 90146 Palermo, Italy

**Keywords:** Obstructive sleep apnea, CPAP therapy compliance, Motivational reinforcement, Emotional support

## Abstract

**Background:**

Obstructive sleep apnea (OSA) is a high prevalence sleep disorder characterized by upper airway obstruction during sleep, nocturnal intermittent hypoxemia, poor sleep quality, risk for cardiovascular and metabolic diseases. The adherence to CPAP is the key for an effective management of these patients.

The aim of the study was to assess the adherence to CPAP therapy with and without early reinforcing interventions, consisting of motivational reinforcement and technical support in the first month of therapy.

**Methods:**

Forty patients with OSA undergoing counseling and a one year follow-up on a quarterly basis were included in the study. Twenty subjects (intervention group) underwent reinforcing interventions with telephone interviews in the first month of therapy, and twenty (control group) remained without reinforcing interventions. The two populations were homogeneous for age, severity of illness and BMI.

**Results:**

During the first month, intervention group patients showed a higher number of nights with a device use ≥4 hours. Average treatment adherence in the first month (days of therapy with at least 4 hours per night on the total number of days from device delivery) was 77.5% in the intervention group and 55.7% in the control group (p = 0.022). At one year the differences between the two groups were not significant.

**Conclusions:**

Our findings suggest that it is important that adequate time and effort is spent to ensure patient comfort at the time of CPAP therapy start to optimize acceptance and adherence to treatment, and suggest that it is necessary to maintain reinforcing interventions over time.

## Background

Obstructive sleep apnea (OSA) is a common sleep disorder characterized by intermittent partial or complete upper airway obstruction during sleep, associated to recurrent arousals, nocturnal intermittent hypoxemia, sleep fragmentation and poor sleep quality. The prevalence of OSA associated with accompanying excessive daytime sleepiness (EDS) is approximately 3 to 7% in adult men and 2 to 5% in adult women in the general population [1]. Factors that increase vulnerability to the disorder include age, male sex, obesity, craniofacial abnormalities, family history, menopause, and behaviours such as cigarette smoking and alcohol use [1].

Sleep-disordered breathing adversely affects daytime alertness and cognition: patients suspected of suffering from sleep apnea show several typical symptoms including habitual snoring (often disruptive to bed partners), feeling unrefreshed at wake-up, EDS or fatigue, lack of concentration, memory impairment, and at times neurobehavioural disturbances [2]. OSA is an independent risk factor for cardiovascular diseases, including hypertension, atrial fibrillation, coronary artery disease, stroke [3], and for metabolic diseases like type 2 diabetes [4]. OSA is associated with automobile accidents [5], reduced participation in work activities, increased absenteeism, and a loss of productivity, with a consequent increase in the use of resources [6]. Severe OSA is associated with increased mortality, especially in young subjects. Although mortality risk associated with OSA tends to disappear from the age of 50, it has been suggested that OSA treatment by continuous positive airway pressure (CPAP) improves survival even in older subjects [7].

CPAP is the gold standard treatment for OSA. Adherence to CPAP treatment is important, since when CPAP is adequately used it eliminates apneas, improves sleep quality and health related quality of life (HRQoL), and reduces EDS [8]. Furthermore, it can reduce morbidity and mortality from cardiovascular diseases as well as consumption of health care resources. However it is not always well tolerated. It requires follow-up, and the adherence rates are often low [8]. Adherence to CPAP therapy is the key for effective management of OSA patients. Epidemiological data show that on average 25% of OSA patients do not accept CPAP treatment and, of those who undertake the therapy, only 30-60% can be considered adherent [8]. An acceptable adherence to therapy is usually considered a minimum of 4 hours/night for at least 70% of the nights of therapy [9]. Some of the key determinants of CPAP rejection and non-adherence may include apprehension regarding how CPAP will make patients look and feel, interference with normal life and sexual functioning, and other behavioural or psychological factors [10]. In order to enhance adherence to CPAP, treatment should be presented as desirable for the patient, must not appear too complex, must be proposed several times, and must be effective.

Several recent studies [11-13] show that adherence to CPAP therapy can be improved with some strategies: patient educational training and information at the start of therapy, timely approach to the resolution of possible causes of non-adherence to therapy, structured follow-up and motivational support. However, the effects in the long-term of a short early motivational support on adherence to treatment are poorly known.

The aim of the study was to assess the adherence to CPAP therapy with and without early reinforcing interventions, consisting of motivational reinforcement and technical support in the first month of therapy, and to evaluate if the possible benefit of the support could extend to one year.

## Methods

Forty consecutive patients (27 male and 13 female) older than 18 years, with diagnosis of OSA and indication to CPAP treatment, according to international guidelines [14,15], were recruited by the sleep disordered breathing Centre of IBIM-CNR Palermo. The total number of 40 patients was chosen a priori. The protocol was approved by the local ethical committee (Azienda Ospedaliera Universitaria Policlinico Paolo Giaccone dell’Università degli Studi di Palermo) and all persons gave their written informed consent prior to their inclusion in the study. Patients with impairments or comorbidities considered likely to interfere with adherence to instructions were excluded: neuromuscular disease, unstable psychiatric disease or cognitive impairment considered likely to interfere with adherence to instructions, myocardial infarction, unstable angina, cardiac failure, cerebrovascular accident, lung disease with awake resting oxygen saturation of less than 90%.

Nocturnal cardiorespiratory recording (Somté or Somnea Compumedics, Abbotsford, Australia) was performed for diagnosis of OSA. On the cardiorespiratory recordings, time between estimated first sleep onset and last morning awakening was analyzed. Apneas and hypopneas were manually analyzed. Apneas were identified on the airflow signal, and were classified as obstructive, central, or mixed, according to behaviour of thoraco-abdominal movements. Hypopneas were scored when a ≥30% reduction in the airflow signal was detected in association with a reduction ≥4% of oxyhemoglobin saturation (SaO_2_) [15]. Apnea/hypopnea index (AHI) was calculated as the number of (apneas + hypopneas)/h of recording that was analyzed. Time with SaO_2_ below 90% (TSat ≤90%) was calculated.

Before CPAP titration, all patients were informed by the medical personnel about the diagnosis with a description of the disease and the consequences of ineffective treatment, and about the follow-up. Then, the nursing staff identified the suitable mask and held a morning session of education and training to the CPAP therapy. The duration of this phase was about 30 minutes. In lab CPAP titration was performed with the AutoSet (ResMed Abingdon UK) auto-titrating CPAP device with full nocturnal polysomnography. Throughout the night and the next morning, the nurses on duty dealt with any discomfort related to the CPAP treatment. Fixed CPAP was prescribed to each patient at the pressure needed to abolish obstructive respiratory events, airflow limitation and snoring, as determined by the overnight AutoSet CPAP titration study. The same CPAP machine was given to every patient (Weinmann SOMNO*comfort* 2e) and all subjects were advised to use their CPAP machine as often as possible when asleep. Then patients were randomised to receive extra early support (intervention group) or standard follow-up (control group).

After receiving the CPAP device, all patients were provided with a telephone number to call the doctor of the sleep centre for support within office hours. The home care provider visited all patients at their home at month 3, 6, 12 from the start of therapy, and each time he downloaded data from the device memory (time of device use per night), controlled BMI, administered Epworth Sleepiness Scale (ESS), and transmitted all data to the sleep centre that analyzed them. In the intervention group ESS was administered also the 30th day from the beginning of the therapy.

**Figure 1:**
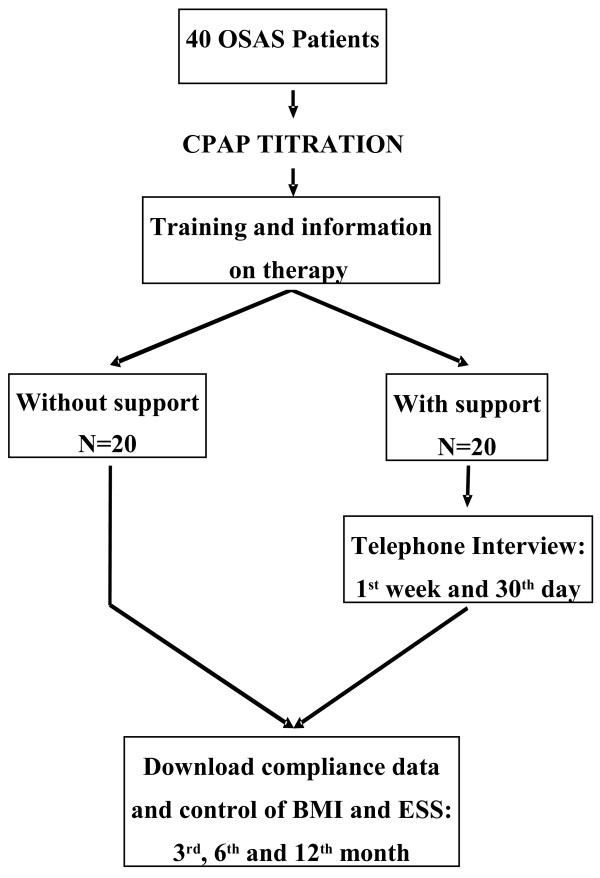
**Flow-diagram showing the path of the proposed support.** OSAS, obstructive sleep apnea syndrome; CPAP, continuous positive airway pressure; BMI, body mass index; ESS, Epworth Sleepiness Score.

Patients in the intervention group received a standardized daily telephone interview from their sleep doctor on the first week of therapy and at one month after therapy start. During the interview they were asked about the most common adverse events during CPAP treatment: i.e. abrasions or rashes, nasal pain, conjunctivitis from air leakage, bloating, sinusitis and rhinitis, difficulty in breathing out, sense of chest tightness, dryness of the nose and mouth, nosebleeds. The doctor reviewed progress and gave advice to manage CPAP-related adverse effects, and encouraged to maintain adherence to therapy. Besides, when necessary, technical support was given by the home care provider. The protocol is shown in Figure 1.

### Statistical analysis

The effect of the support on CPAP adherence, defined as the monthly average number of days of therapy ≥4 hours, was evaluated by non parametric Wilcoxon/Kruskal-Wallis test. Data were reported as mean ± SD. A p <0.05 was considered significant. Statistical analysis was performed by commercial software (JMP 8.0 SAS Institute Inc.).

## Results

The anthropometric and clinical data of the study sample are shown in Table 1. The two groups were homogeneous for age, severity of illness, TSat ≤90%, BMI, daytime sleepiness assessed by ESS. Reinforcing intervention patients used CPAP no longer compared to control group (mean 4.3 hr/night vs 3.8 hr/night and median 5.1 hr/night vs 4.5 hr/night) in one year.

**Table 1:** Subjects baseline characteristics

**Study**	**Intervention group (n. 20)**	**Control group (n. 20)**	**p**
**AGE (yr)**	58.55 ± 13.2	55.65 ± 8.25	ns
**AHI (n/h)**	44.05 ± 16.90	44.45 ± 25.18	ns
**BMI (kg/m**^**2**^**)**	33.93 ± 5.44	34 ± 5.99	ns
**ESS**	8.95 ± 5.74	10.55 ± 6.21	ns
**TSat ≤90%**	20.88 ± 15.22	20.77 ± 22.03	ns

AHI (apnea-hypopnea index), BMI (body mass index), ESS (Epworth Sleepiness Score); TSat ≤90% (% of study time with oxygen saturation below 90%).

In the intervention group, one subject was not available at the 12th month of follow-up. In the control group, one subject was not traceable from the 3rd month, and another one at 12th month of follow-up.

Complaints of the patients during the first month were addressed in real time with immediate action. The most frequent complaints regarded nose and mouth dryness: this problem was fixed with replacement of the CPAP humidifier in two cases, and with replacement of nasal with full face mask in 3 subjects. In two cases topical products for abrasions due to the mask were prescribed. The level of the therapeutic pressure was reduced of 1 cm H_2_O in 2 patients for reported sense of excessive pressure. During the first month of treatment with support, one patient was not adherent to therapy for nocturnal xerostomia that did not improve either with an increase of the heat humidifier or after replacement of the nasal mask with a face mask. Patients of the intervention group showed a higher number of nights with a device use ≥4 hours (23.2 days in the intervention group vs 16.0 for the control group, p = 0.022). Average treatment adherence was 77.5% in the intervention group and 55.7% in the control group.

**Figure 2:**
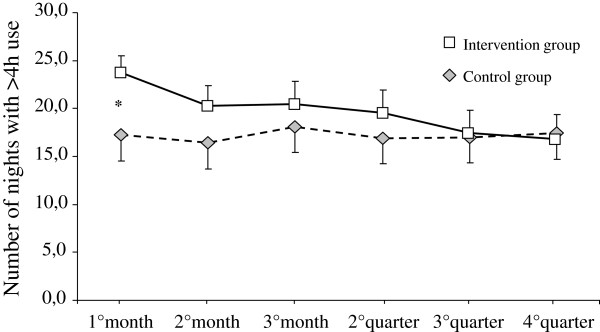
**Average treatment adherence during the follow-up: mean (±SE) monthly number of nights with use >4 hours for intervention group and control group.** The asterisk indicates a statistically significant difference between two means.

The difference in adherence became non-significant at the 2^nd^, 3^rd^ month and at the 2^nd^, 3^rd^ and 4^th^ quarter (Figure 2). At second quarter compliance was 66.7% in the intervention group vs 58.3% in the control group (p = NS), and at fourth quarter adherence was 54.3% in the intervention group vs 56.3% in the control group (p = NS). Both in the intervention and in the control group, daytime sleepiness measured by the ESS was lower at the 3^rd^, 6^th^ and 12^th^ month than at baseline with no significant differences between the two groups (Table 2). The reduction of adherence through time covered all the patients equally, irrespective of initial ESS.

**Table 2:** Epworth sleepiness score during follow-up

**Study**	**ESS baseline**	**ESS 1°month**	**ESS 3°month**	**ESS 6°month**	**ESS 12°month**
**EES intervention group**	8.95 ± 5.74	3.6. ± 2.43	4.35 ± 3.34	5.05 ± 4.31	5.68 ± 4.02
**EES control group**	10.55 ± 6.21		5.10 ± 3.32	4.16 ± 2.14	4.00 ± 2.82

Daytime sleepiness measured by the ESS (Epworth Sleepiness Score), baseline, at the 3^rd^, 6^th^ and 12^th^ month.

## Discussion

Although many patients with OSA benefit from treatment with CPAP, a significant proportion of them do not initiate or drop therapy. CPAP therapy adherence is highly multifactorial: from medical and physiological factors, to equipment-related side effects and technical factors. A wide variety of approaches might improve CPAP adherence in the real world: education to CPAP, additional support, telemedicine approaches, use of hypnotic sleep aids, cognitive behavioural treatments [16]; among possible interventions to enhance CPAP use, we used telephone support, increased interaction with sleep provider, planned follow-up. The aim of the study was to assess the effect of a short time reinforcing intervention on the adherence to CPAP therapy in the months following interruption of the support. The assistance consisted of motivational reinforcement, technical and clinical support on the first month of therapy. The activated support led to better adherence to therapy in terms of number of nights with use ≥4 hours during the first month. However the benefits of the support during the first month diminished later and did not extend to one year.

Like other studies [17,18], we used a type of intervention and support only in the initial phase of therapy with standardized telephone calls during the first week and on the 30th day from delivery of the device. During the phone contact, medical staff supported the patient process of acceptance of the chronic therapy, with the assistance of the home care provider at home. Some studies have shown that early low adherence to therapy is a feature of patients quitting treatment in the first year of use. Mc Ardle et al. [19] studied 1,103 patients with a median follow-up of 22 months and found that compliance at 3 months was strongly predictive of long-term use.

Broström et al. [10] analyzed the facilitators and barriers to CPAP adherence in a sample of 23 patients and concluded that emotional and practical shortfalls support over the first days of therapy do not improve adherence. A more prolonged intervention may be necessary to keep for as long as possible the effect of the additional support. Hoy et al. [20] clearly showed for the first time that an intensive support ended in an improvement of the therapeutic observance of the CPAP over 6 months, with greater improvement in OSA symptoms, mood, and reaction time. In this study, unlike in our studies, the bed partners were actively involved in the initial phase of education to CPAP, three nights CPAP titration in the sleep center were performed, a home visit at the 14th day and at the beginning of the fourth month therapy was made. Probably, these factors were important reinforcing elements in the long-term adherence to CPAP therapy [20].

Lewis et al. [17] recruited in a sleep centre seventy-two consecutive patients starting CPAP for OSA who were randomised to receive standard follow-up or extra early support. Patients had further appointments with a sleep physician at sleep centre at 1 month, 6 months and 12 months. However, almost a third of patients in the control group failed to attend at 6 months, so that reliable data on CPAP use, ESS and side-effects were lacking. In contrast to this and other studies, our protocol actively involved an home care provider in the immediate management and resolution of patients’ problems. This allowed us to accurately obtain the adherence data, unlike in other studies [17] where an active participation of the patient for data collection was expected.

Another randomized, controlled, parallel study about effects of basic vs augmented CPAP education and support, based on a sample of 108 patients of the Chinese population, assessed outcomes of extra early CPAP education and support. The authors analyzed quality of life by Calgary sleep apnea quality of life index, and cognitive function after 1 month and 3 months: unlike in our study, augmentation of CPAP education and support did not increase CPAP compliance, but led to a greater improvement in quality of life during the reinforced period [18].

Attitudes and beliefs as well as early problems and poor initial CPAP usage are strongly predictive of long-term poor adherence [21-24]. Consequently, early interventions targeting issues surrounding CPAP knowledge, benefits and expectations, the initial novel experience, and common problems in achieving sleep with CPAP may be substantially alleviated by timely education and clinical management [9]. Adequate early support is of major importance for addressing psychosocial and behavioural barriers establishing effective CPAP treatment; the early management of technical problems is clearly important to improve compliance [13,25].

Aloia et al. [26] applied time series analysis to examine the differences in individual adherence to CPAP therapy on a sample of 70 OSA subjects and identified 7 different categories of adherence: good users, slow improvers, slow decliners, variable users, occasional attempters, early drop-outs and non-users. Although CPAP adherence is generally established early, two subjects of their groups (slow improvers and slow decliners) developed new, yet consistent, use patterns later into therapy. Their results suggest that two reinforcing intervention times may be relevant when targeting CPAP adherence: in the early course of treatment and at 3 to 6 months of treatment. Aloia’s study results, and the inefficacy of our early support alone, show that the focus on adherence should not be withdrawn after the first month.

Strengths of our study are its randomised, controlled design, and the active participation of the home care provider with the sleep center. A weakness is a single center study with a relatively modest number of patients and the lack of additional support over the first month. Nevertheless, it allowed us to demonstrate a significant effect of the support program. Repeating these results in other studies would be relevant to confirm the benefit.

## Conclusions

Our findings suggest that it is important that adequate time and effort is spent to ensure patient comfort when CPAP therapy is initiated to optimize acceptance and adherence to treatment, and suggest that it is necessary to maintain reinforcing interventions over time. Although we can measure side effects and improve re-attendance, more work is necessary to tailor interventions for early identification of poor users, and especially to look out for patients who do not re-attend at all.

Our experience has confirmed the benefit of a medical, motivational and technical support in the first month. However it was not effective in the long term, suggesting that patients treated with CPAP need to maintain a reinforcement beyond the first month of therapy.

## Abbreviations

CPAP: Continuous positive airway pressure; EDS: Excessive daytime sleepiness; ESS: Epworth sleepiness scale; HRQoL: Health related quality of life; OSA: Obstructive sleep apnea.

## Competing interests

The authors have no conflict of interest to disclose.

## Authors’ contributions

LBA contributed to design the study, was responsible for collection of data and for their organization in a data base, provided the support in the first month of therapy and drafted the manuscript. SA conceived the study, collected baseline data, contributed to the interpretation of the data and drafted the manuscript. IIS collected baseline data, contributed to the organization in a data base and to critical revision of the article. MO collected baseline data, contributed to design the study, contributed to the interpretation of the data and to critical revision of the article. RS performed the statistical analysis, contributed to the interpretation of the data and to critical revision of the article. IG conceived the study, collected baseline data, contributed to the interpretation of the data and performed critical revision of the article. All Authors actively discussed the subject, revised the paper, and provided final approval.

## Pre-publication history

The pre-publication history for this paper can be accessed here:

http://www.biomedcentral.com/1471-2466/14/78/prepub
